# Simple Fracture of the Lower Jaw

**Published:** 1887-10

**Authors:** William Daniel Saunders


### Simple Fracture of the Lower Jaw.

BY WILLIAM DANIEL SAUNDERS, D.D.S.

The term fracture signifies a solution of the continuity of a bone. As to their nature, fractures are divided into four classes: simple, compound, comminuted, and complicated. They are also designated as transverse, longitudinal, or oblique, according to the direction in which the fracture extends.

A simple fracture is one in which the bone is broken once in two, and having neither of its fragments exposed to the air.

A compound fracture indicates a condition in which the bone is divided, together with the tissues and integuments, thus allowing the ends of the bone to protrude.

A comminuted fracture signifies that the bone has been broken into more than two pieces, while any of the above may be classified as complicated if in addition to the bone being broken any important organ, blood-vessel, or nerve is injured.

The seat of the fracture in a large proportion of cases is found to be in the body of the jaw, and rarely situated in the rami or at the symphysis, since if any bone is brought to bear on the jaw at this point it is immediately transmitted to the body, the symphysis acting as a key-stone to an arch. The rare occurrence of fractures in the rami is attributed to the fact that these are much more readily dislocated than broken.

As a result of the breaking of the lower jaw we generally have a complicated fracture, which is made so by the dislodgement of the teeth and the injury which results to the inferior dental artery, vein, and nerve. The structures last mentioned are rarely ruptured by a fracture to the lawer jaw, but they may be quite severly stretched and irritated.

The causes of fractures in this locality are quite completely enumerated in the following: blows, falls, gun-shot wounds, and the extraction of teeth, although the latter cause rarely injures any thing but the alveolus, except perhaps in those cases where the organic constituents of the jaw are greatly diminished in amount.

First and foremost among the signs and symptoms of a fractured condition of the lower jaw is deformity. This may consist in the symphysis pointing towards the side upon which the fracture occurs, which condition is due to the contraction of the muscles which cause the fractured ends to be pulled by each other. This is a strong differential point by which fractures may be distinguished from dislocation, for in the latter the symphysis always points toward the side opposite the dislocation.

Another prominent sign of fracture is found in the increased mobility of the part, that is, the part is susceptible to a larger range of movement.

Crepitis is a symptom by which your diagnosis may be confirmed. By this term is understood that peculiar, characteristic, grating sensation, which can be felt or heard when the ends of a fractured bone are rubbed together.

Salivation and laceration of the gums generally accompany fracture of the lower jow.

Salivation means an abnormally increased flow of saliva. This condition is brought about by the injury causing a reflex stimulation of the salivary glands. The laceration of the gums which follows a fractured condition is due to the protrusion of the ends of the broken fragments.

When the signs and symptoms indicate a fracture of the jaw, the next thing which would naturally suggest itself would be its treatment. This is all comprised under the propositions, "replace and keep in place." This is found to be simple in theory, but very difficult in practice, as the tendency here is to have the fragments drawn out of place by the action of the muscles.

In reducing a fracture of the lower jaw one of the first things to consider is the retention of fragments. If the teeth or fragments of bone are wholly displaced at the seat of fracture, and especially if the teeth be molars, it is not wise to replace them, but on the contrary they should be removed. In this way we prevent the severe inflammation and non-union which might otherwise follow. But if the teeth or fragments are but partially displaced they may be replaced, if they do not interfere with the proper adjustment of the parts.

If the case in hand is a simple fracture, replace the fragments to as near the normal condition as possible, always using as a guide the unfractured side. In replacing the fragments always be careful and not injure the part by using too great freedom in manipulating, as severe inflammation and non-union might result. Having the fragments replaced to a normal condition, the next indication to be met would be to securely retain them in place until they become united. To accomplish this several methods have been employed, as wireing the teeth together, drilling through the fragments, passing a silver wire through and twisting on the inside of the jaw, or by using splints, such as pasteboard, sole leather, or gutta percha, held in place by a four-tailed bandage.

Wireing the teeth together is seldom employed, except in cases of non-union. The objections to it are that the tendency is to pull the fragments together at the top or near the teeth and separate them at the bottom, or the teeth may be moved, and the desired object thus defeated.

Drilling through the fragments and passing a silver wire through is a proceedure not much employed at present, except in those cases where there is a tendency to non-union, or in case the patient becomes delirious.

The use of splints has almost entirely replaced the other methods of holding the fragments in place. As was stated previously, these may be made of pasteboard, sole leather, or gutta percha, cut to the desired shape, softened by placing in cold or hot water, molded to the part, and held in position by a bandage. The four-tailed bandage seems to meet the indications better than any other.

As to the exact time of union nothing definite can be said, taking as a general rule from four to six weeks, during which time the patient should lie on his back and take liquid food; always avoiding to masticate any thing hard. The teeth of the lower jaw should be kept in close contact with those of the upper jaw. The observation of the above precautions tends to prevent any displacement of the parts.

The union of fractures is accomplished by the injury acting as an irritant, causing more blood, and consequently more food, to be thrown to the part. A plastic deposit now takes place all around the sides of the fragments. This serves to hold them in place. Later the ends of the fragments revert to the embryonal state, and a plastic deposit also occurs here. This plastic deposit at first is of a jelly-like consistance; later it begins to resemble cartilage. This state is succeeded by small deposits of bony material in this cartilage, which finally becomes entirely replaced by bone. Now a process of absorption is set up, and in the course of a year or so the callous, as it is called, almost entirely disappears.

